# High yields of hydrogen production from methanol steam reforming with a cross-U type reactor

**DOI:** 10.1371/journal.pone.0187802

**Published:** 2017-11-09

**Authors:** Shubin Zhang, Yufeng Zhang, Junyu Chen, Xuelin Zhang, Xiaowei Liu

**Affiliations:** 1 MEMS Center, Harbin Institute of Technology, Harbin, China; 2 Key Laboratory of Micro-Systems and Micro-structures Manufacturing, Ministry of Education, Harbin, China; Universita degli Studi della Tuscia, ITALY

## Abstract

This paper presents a numerical and experimental study on the performance of a methanol steam reformer integrated with a hydrogen/air combustion reactor for hydrogen production. A CFD-based 3D model with mass and momentum transport and temperature characteristics is established. The simulation results show that better performance is achieved in the cross-U type reactor compared to either a tubular reactor or a parallel-U type reactor because of more effective heat transfer characteristics. Furthermore, Cu-based micro reformers of both cross-U and parallel-U type reactors are designed, fabricated and tested for experimental validation. Under the same condition for reforming and combustion, the results demonstrate that higher methanol conversion is achievable in cross-U type reactor. However, it is also found in cross-U type reactor that methanol reforming selectivity is the lowest due to the decreased water gas shift reaction under high temperature, thereby carbon monoxide concentration is increased. Furthermore, the reformed gas generated from the reactors is fed into a high temperature proton exchange membrane fuel cell (PEMFC). In the test of discharging for 4 h, the fuel cell fed by cross-U type reactor exhibits the most stable performance.

## Introduction

Fuel cell technology, a promising means of converting chemical energy to electrical energy, has been regarded as one of the solutions to energy crisis due to the advantages of high efficiency, low emission, silent operation, environmental friendliness and sustainability [1–3]. The proton exchange membrane fuel cell (PEMFC) is particularly attractive and promising for portable applications because of its simplicity in design and operation, mild operating conditions and ability to provide high power density [4–6]. One of the major problems of portable PEMFCs is the difficulty involved with the storage and handling of pure hydrogen. In this situation, on-board hydrogen supplying as methanol steam reforming (MSR) received so much attention because of easy integration with PEMFC, especially high temperature (HT) PEMFC [7–9].

In the process of MSR, methanol and water are vaporized and fed into reformer, where the chemical reaction takes place and hydrogen is produced. The reaction for MSR is:

$${\mathrm{CH}}_{3}{\mathrm{OH}+H}_{2}O\to {\mathrm{CO}}_{2}{+3H}_{2}$$ (1)

Using Cu-based catalyst, carbon monoxide is inevitably generated. Reactions of methanol decomposition and reverse water gas shift reaction are commonly used to express the mechanism [10].

$${\mathrm{CH}}_{3}\mathrm{OH}\to {\mathrm{CO}+2H}_{2}$$ (2)

$${\mathrm{CO}}_{2}{+H}_{2}\to {\mathrm{CO}+H}_{2}O$$ (3)

Since reforming is an endothermic reaction and must be activated above at least 180°C, extra heat must be supplied. Thus, a micro reformer should be integrated with a combustor where heat is generated from methanol/air or hydrogen/air catalytic combustion or even electrical power. The combustion reactions are:

$${\mathrm{CH}}_{3}\mathrm{OH}+3{/2O}_{2}=2H_{2}{O+\mathrm{CO}}_{2}$$ (4)

$$H_{2}{+1/2O}_{2}{=H}_{2}O$$ (5)

Recent studies have proposed different structural reformers integrated with combustor for the reforming process. Efforts were made both numerically and experimentally. For instance, Hsueh et al. [11] presented an analysis on 3D modeling of a plate methanol steam micro-reformer and a methanol catalytic combustor with parallel flow fields and serpentine flow fields. The simulation results revealed that the methanol conversion of the micro-reformer with the serpentine flow field and the combustor with the serpentine flow field has the best performance among all the possible combinations. Similarly, annulus reactors were designed and simulated by Chein et al. [3, 12]. The studies indicated that effective heat transfer characteristics in mini-scale reactors are essential to higher hydrogen productivity. Chein et al. [13] also conducted experimental studies on an integrated compact reactor consisting of a vaporizer, a reformer and a combustor to identify the flow and heat transfer effects on the reactor performance. Three different types of reformers, namely patterned microchannel, single plain channel and inserted catalyst layer were fabricated. At last, microchannel reformer exhibited the highest methanol conversion among the reactors. In these designs, the main idea of increase reforming performance was to enhance heat transfer efficiency, which helps build up low gradient temperature distribution in the micro reformers. On the other hand, uniform flows were found to be an important factor in microreactors for achieving high performance [14–16]. Therefore, novel reactor structure was proposed to improve both flow distribution and heat transfer. Chein et al. [17] and Suh et al. [18] applied this idea in tubular reactors. Chein et al. presented miniature reactors with thin baffle plates installed inside the catalyst beds to disturb the reactant flow. The simulation results showed that the temperature reactant flow and catalyst bed can be increased, which led to improved methanol conversion. In addition, the pressure drop across the reactor was found to be less significantly influenced by the baffle plates in miniature scale reactors. Suh et al. carried out experiments on an internally heated reformer under different operating conditions to show the advantage over externally heated reformer. When integrated with HT-PEMFC, methanol steam reformer were usually heated by burning the un-utilized hydrogen in the anode exhaust. In the work of Kim et al. and Besser et al. [19, 20], hydrogen combustion showed the ability of generating sufficient amount of heat to sustain the steam reforming reaction. Moreover, the system became totally pollution-free in this way (the anode exhaust contains carbon monoxide) and energy efficiency was also increased. Literature review suggests that, on the credit side, most of the numerical results on microreactors put forward appealing results as they briefly found and made use of one or two strong points. However, more accurate and precise experiments are needed to realize the fabrication of complex structural microreactors and then validate new ideas.

Based on the understanding of micro-reformers integrated with combustors, this study presents a numerical and experimental investigation on the performance of a cross-U type microreactor which contains a reformer and a combustor. A three-dimensional model is established to analyze the mass flow and temperature characteristics. The simulation results exhibit the temperature distribution across the whole reactor and the methanol mass fraction in the reformer. In order to validate the simulation results, Cu-based microreactors with high thermal conductivity have been designed, fabricated and tested. At last, the reformed gas was fed directly into a HT-PEMFC for stability test.

## Numerical

### Model description and assumptions

**Fig 1:**
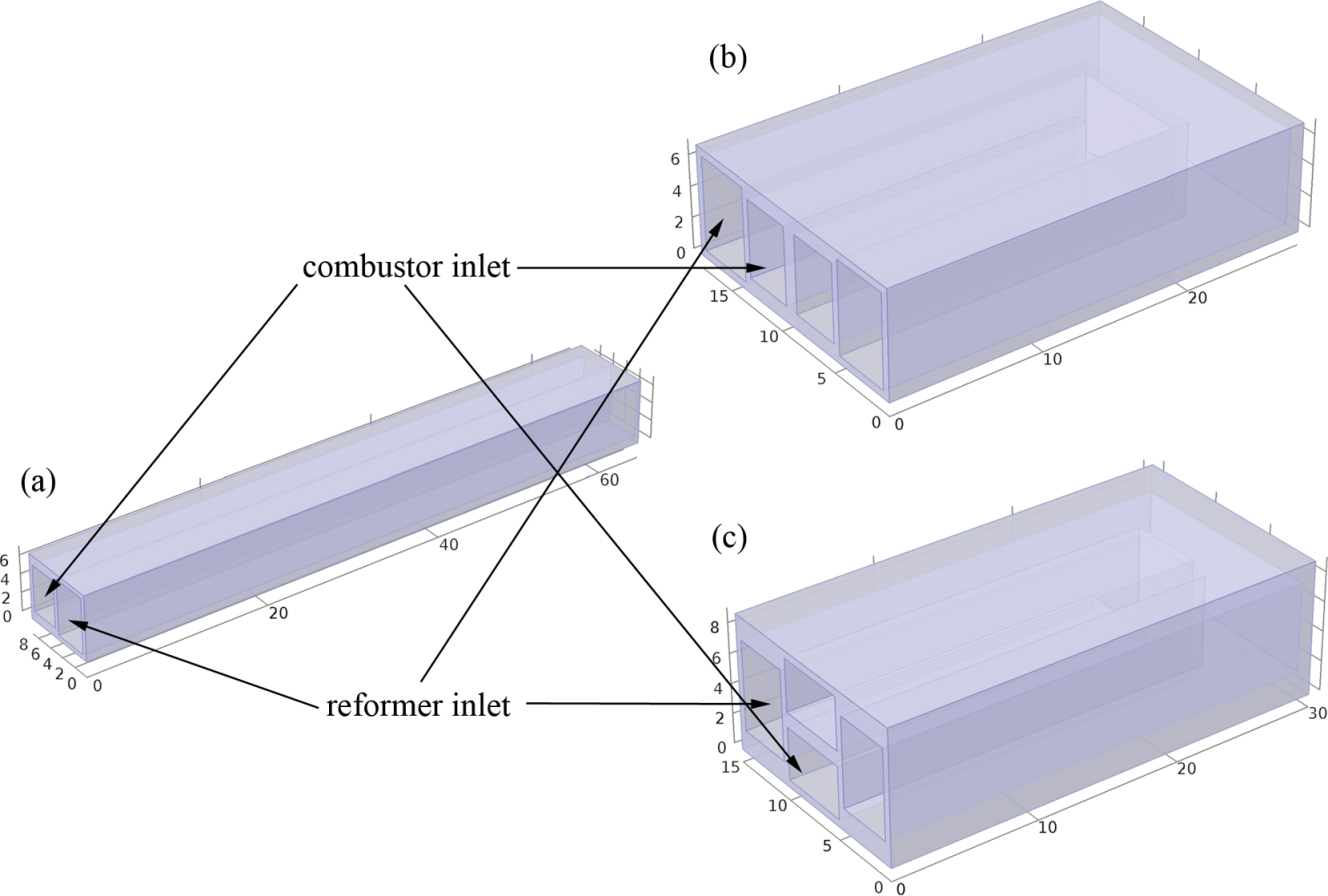
Geometry of the reactors in the simulation: (a) tubular, (b) parallel-U, and (c) cross-U.

Fig 1 illustrates the Geometry of the presented reactors. Fig 1A shows a tubular reactor with rectangular cross-section. The cross-sectional area of reformer is 4 × 6 mm, and that of combustor is 3.5 × 5 mm. Fig 1B and 1C show the parallel-U and cross-U type reactors, respectively. Length of reformer is 6.5 cm for each reactor while that of combustor is a little shorter for parallel-U and cross-U type reactors. All the reactors are covered with thermal insulating jacket, of which the thickness is neglected. In this section, methanol steam reforming in the reformer and hydrogen/air combustion in the combustor are simulated simultaneously.

The mass and momentum transport phenomena in the catalyst beds and energy transport in the whole reactor are described by Maxwell-Stefan diffusion equation, Darcy’s Law, and some other non-linear partial differential equations. For the sake of simplicity, the following assumptions are made. Parameters used in the 3D model are listed in Table 1.

1. All gases are ideal gases and are weakly compressible, steady-state and laminar.
2. In the inlet of reformer, methanol and steam are pre-heated to 240°C, ready for reforming reaction.
3. Catalyst beds in both reformer and combustor are treated as a porous medium.
4. Specific heat capacity, viscosity and thermal conductivity for hot gas are regarded as constant.

**Table 1:** Parameters used in the 3D model.

Parameter	Value	Reference
Activation energy for MSR (J mol^-1^)	1.09 × 10^5^	[11]
Activation energy for rWGS (J mol^-1^)	1.15 × 10^5^	[11]
Activation energy for MD (J mol^-1^)	1.42 × 10^5^	[11]
Activation energy for HO (J mol^-1^)	2.12 × 10^4^	[11]
Pre-exponential factor for MSR	9.55 × 10^12^	[11]
Pre-exponential factor for rWGS	1.65 × 10^13^	[11]
Pre-exponential factor for MD	1.65 × 10^13^	[11]
Pre-exponential factor for HO	5 × 10^14^	[11]
Hot gas specific heat (J kg^-1^ K^-1^)	1	[17]
Hot gas viscosity (kg m^-1^ s^-1^)	3 × 10^−5^	[17]
Porosity of catalyst bed in reformer	0.35	[17]
Porosity of catalyst bed in combustor	0.4	[17]
Permeability of catalyst bed (m^2^)	2.379 × 10^−12^	[17]
Average mass diffusion coefficient (m^2^ s^-1^)	6.8 × 10^−5^	[17]
Thermal conductivity of the hot gas (W m^-1^ K^-1^)	0.04	[17]
Thermal conductivity of reactor substrate (W m^-1^ K^-1^)	401	-
Thermal conductivity of the catalyst (W m^-1^ K^-1^)	20	-
Heat transfer coefficient for reactors (W m^-2^ K^-1^)	300	-
Heat transfer coefficient for atmosphere (W m^-2^ K^-1^)	30	-
Ambient temperature (°C)	25	-

### Governing equations

The mass transport in reformer and combustor can be described using the Maxwell-Stefan diffusion equation:

$$\nabla \left(\rho \omega_{i}u-\rho \omega_{i}\sum_{j=1}^{n}D_{ij}\left(\nabla x_{j}+\left(x_{j}+\omega_{j}\right)\frac{\nabla p}{p}\right)\right)=R_{i}$$ (6)

The flows of gaseous species through the catalyst beds are described by Darcy’s Law:

$$\nabla \left(\rho \left(-\frac{\kappa }{\eta }\right)\nabla p\right)=0$$ (7)

In the above equations, *ρ* denotes the gas density, *ω* the mass fraction, **u** the flow field velocity, *D* the diffusivity, *R* the reaction rate, *η* the viscosity, *κ* the permeability of the porous catalyst beds, and *p* is the pressure in either reformer or combustor.

The energy transport in the porous beds of both combustor and reformer can be described by:

$$\nabla \cdot \left(-k_{\mathrm{eff}}\nabla T\right)+\rho C_{p}u\cdot \nabla T=Q$$ (8)

where *C*_*p*_ represents the heat capacity of gas flows in reformer or combustor, *k*_eff_ is the modified effective thermal conductivity of the porous medium filled with gas, *Q* represents the heat source. Heat source in reformer due to reaction is given by:

$$Q=\sum_{i=1}^{3}\Delta H_{i}\cdot r_{i}$$ (9)

Where Δ*H* is the reaction enthalpy, *r* stands for the reaction rate of Eqs (1) (2) and (3), namely *r*_MSR_, *r*_MD_, and *r*_rWGS_. Similarly, the reaction rate of H_2_/air combustion is represented by *r*_HO_. The modified effective thermal conductivity for catalyst bed is given by:

$$k_{\mathrm{eff}}=\varepsilon k_{g}+\left(1-\varepsilon \right)k_{s}$$ (10)

Where *ε* stands for the porosity, *k*_g_ and *k*_s_ are the thermal conductivities for the gas and solid phases. In this study, the Arrhenius equation [21] is employed to calculate chemical reaction rate coefficients. They can be expressed as follows:

$$r_{\mathrm{MSR}}=k_{1}C_{\mathrm{MeOH}}^{0.6}C_{H_{2}O}^{0.4}\exp\left(-\frac{E_{1}}{RT}\right)-k_{-1}C_{H_{2}}C_{{\mathrm{CO}}_{2}}\exp\left(-\frac{E_{1}}{RT}\right)$$ (11)

$$r_{\mathrm{MD}}=k_{2}C_{\mathrm{MeOH}}^{1.3}\exp\left(-\frac{E_{2}}{RT}\right)$$ (12)

$$r_{\mathrm{rWGS}}=k_{3}C_{{\mathrm{CO}}_{2}}^{}C_{H_{2}}^{}\exp\left(-\frac{E_{3}}{RT}\right)-k_{-3}C_{\mathrm{CO}}^{}C_{H_{2}O}^{}\exp\left(-\frac{E_{3}}{RT}\right)$$ (13)

$$r_{\mathrm{HO}}=k_{4}C_{H_{2},\mathrm{combustor}}\exp\left(-\frac{E_{4}}{RT}\right)$$ (14)

where *E*_*i*_ stands for activation energy for reaction *i*. *k*_1_, *k*_2_, *k*_3_ and *k*_4_ are the forward rate constants, and *k*_−1_ and *k*_−2_ are the backward rate constants for the reversible reactions.

### Boundary conditions

The inlet gas compositions are constant. The mole ratio of H_2_ and O_2_ is 5:3 for combustor and methanol to water ratio (MWR) is 1:1.2 for reformer. At the interface between the flow channel and the solid wall, there is no slip and fluxes. Inside the porous area of catalyst beds, the velocities, species concentrations and species fluxes are continuous. The inlet temperature is 240°C for both reformer and combustor. Inside each simulation subdomain, temperature is continuous. At the overall surface of the integrated reactor, heat flux from the insulating jacket to the surroundings is given by:

$$q=h_{j}\left(T_{\mathrm{sub}}-T_{\mathrm{amb}}\right)$$ (15)

The heat exchange between the catalyst beds and copper substrate is described by:

$$q=h_{t}\left(T-T_{\mathrm{sub}}\right)$$ (16)

In the above equations, *h*_*j*_ and *h*_*t*_ are the heat transfer coefficient, *T* is the temperature for reformer or combustor, *T*_sub_ is the copper substrate temperature, and *T*_amb_ is the ambient temperature.

### Simulation results and discussion

All of the coupled governing equations with the boundary conditions were solved simultaneously using COMSOL Multiphysics. Four modules, general heat transfer, Darcy’s Law and transport of diluted and concentrated species were applied for solving the set of equations. A refined mesh were used in the boundary layers, interface and corner regions where the variable gradients are prominent. Finer meshes were used in other regions near the inlet and outlet. To ensure numerical convergence and solution accuracy, over 40000 meshes were generated. Degrees of freedom solved for were 336243, 499838, and 513795 for tubular, parallel-U, and cross-U type reactors, respectively. The simulation process took about 20 to 30 min for each reactor on an Intel Core^TM^ i5-2300 computer with 8 G memory.

**Fig 2:**
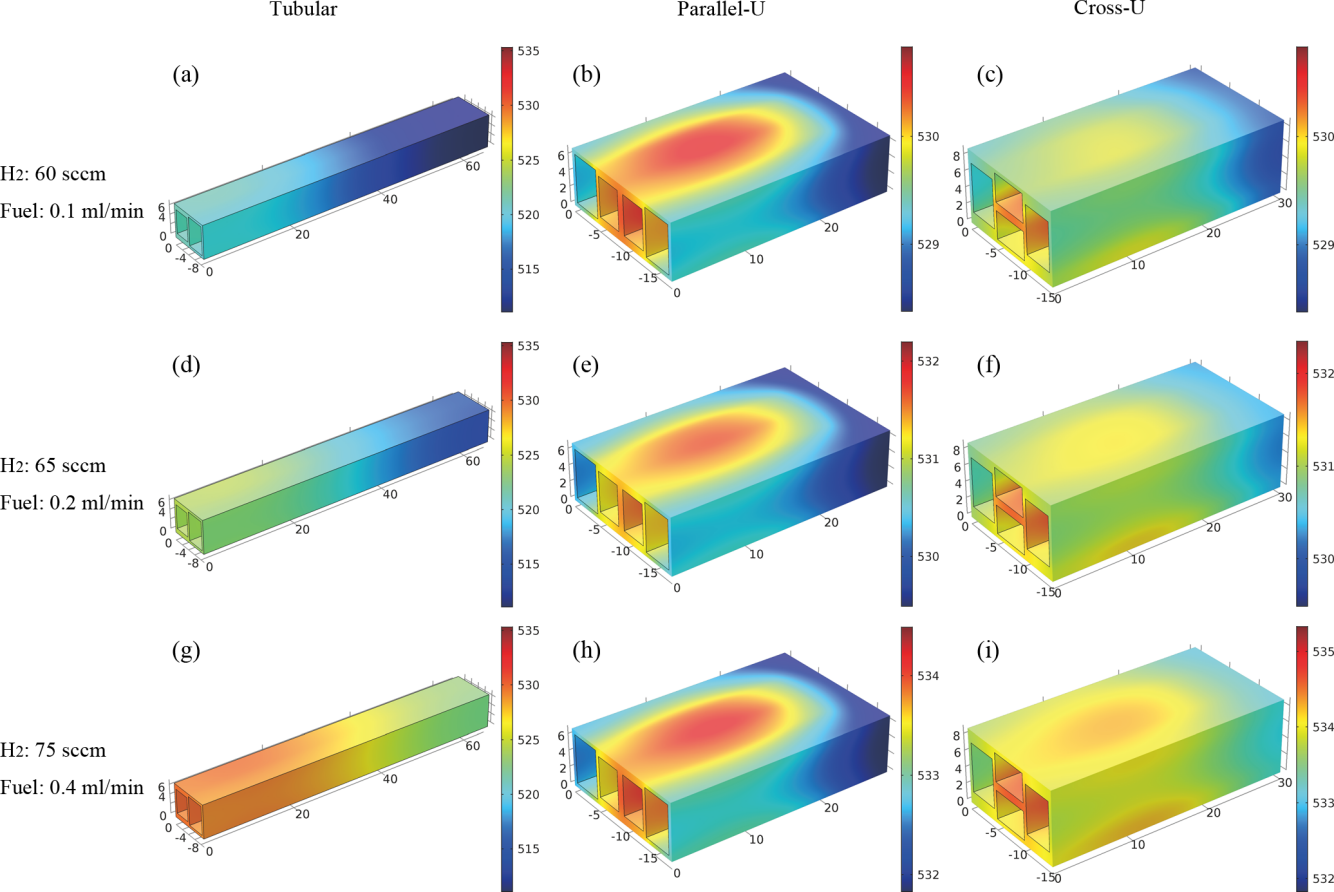
Simulation results of temperature distribution in the reactors with different fuel feeding rates.

Fig 2 shows the simulation results of temperature distribution in the reactors. It is supposed that methanol solution with a MWR of 1:1.2 was already vaporized and then fed into reformers. The liquid flow rates were 0.1, 0.2, and 0.4 ml min^-1^. Accordingly, H_2_ flow rates for combustion were set as 60, 65, and 75 sccm. It is easy to find that the temperature of tubular reactor is the lowest among the three. This can be explained by the reactor structure itself. Table 2 lists the geometry data of the three reactors. The lengths and cross-section areas of the reformers are all the same. Therefore, the internal surface areas of the reformers are the same. Combustor length of the tubular reactor is the biggest among the three, making its combustor’s internal surface area the largest. Obviously, larger internal surface created more interfacial area for heat conduction between hot gas and reactor substrate. However, the external surface area of the tubular reactor is also the largest, which results in severe heat dissipation into the ambient and predominantly makes the temperature of the tubular reactor the lowest. Comparing the cross-U type reactor to the parallel-U type reactor, the internal surface area of its combustor is larger and the external surface area is smaller. Both of factors lead to a better performance of the cross-U type reactor. On one hand, dividing the pictures in Fig 2 into longitudinal comparison, temperature of the reactors were increased with more hydrogen supplied. As a result, the reforming reaction was enhanced so that higher methanol conversion can be acquired when feeding higher rate of methanol solution. On the other hand, a crosswise comparison of Fig 2B and 2C clearly shows that the temperature difference across the cross-U type reactor is less than that across the parallel-U type reactor. With the same color legend, the same pattern was found comparing Fig 2E and 2F and Fig 2H and 2I. It indicates that temperature are more uniformly distributed in cross-U type reactor. In Fig 2D, 2E and 2F, simulation data shows the temperature difference in cross-U reformer is 1.9°C, while that is 2.8°C in parallel-U and 6.2°C in tubular reactor. As a result, the cross-U structure made the best use of the heat generated from hydrogen combustion.

**Table 2:** Geometry data of the three reactors.

Geometry data\Reactor type	Tubular	Parallel-U	Cross-U
Length of reformer (mm)	65	65	65
Length of combustor (mm)	65	48	51
Cross-sectional area of reformer (mm^2^)	24	24	24
Cross-sectional area of combustor (mm^2^)	17.5	17.5	17.5
Internal surface area of reformer (mm^2^)	1300	1300	1300
Internal surface area of combustor (mm^2^)	1105	816	867
External surface area (mm^2^)	2123	1661	1627

**Fig 3:**
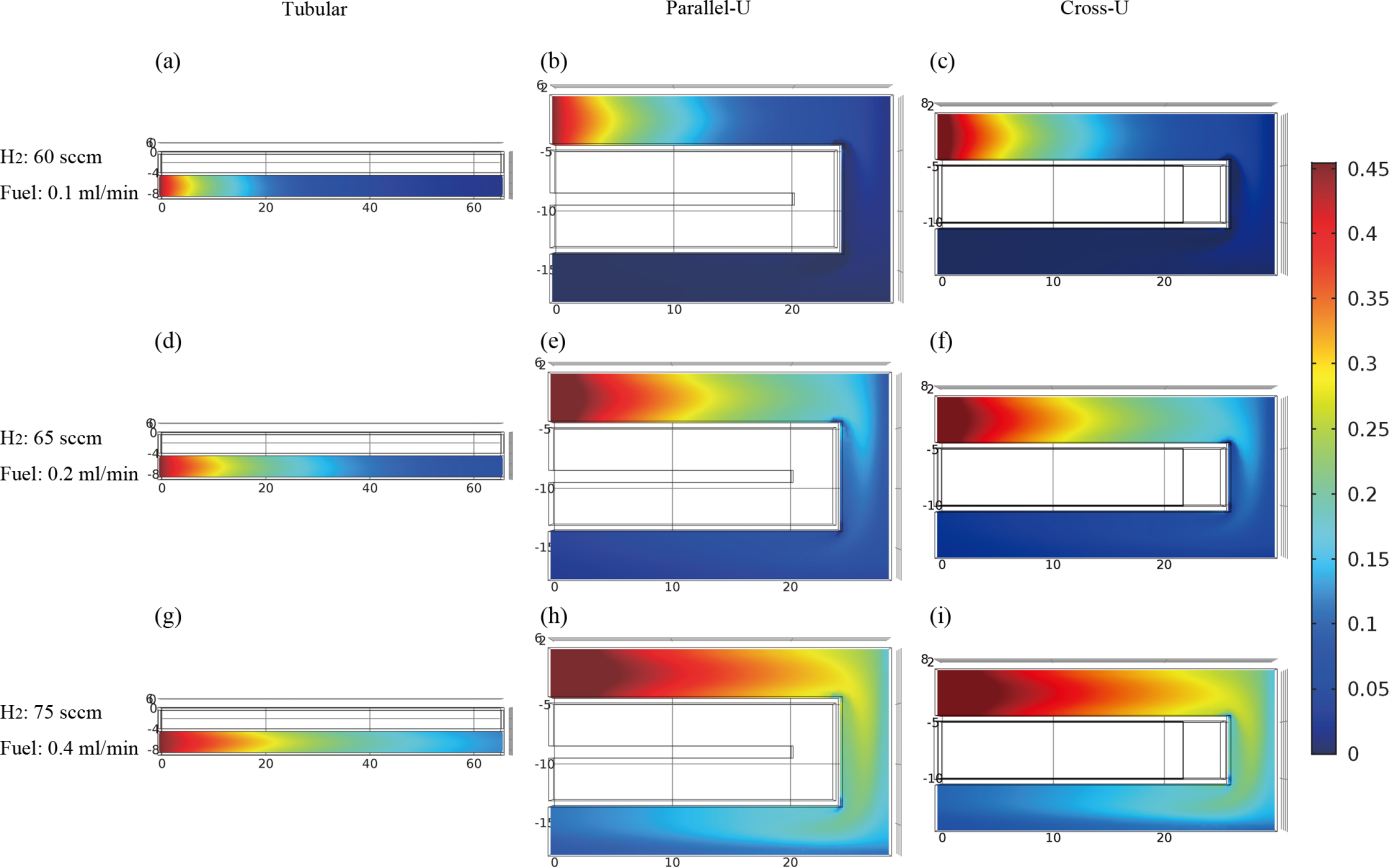
Simulation results of methanol mass fraction in the reformers with different fuel feeding rates.

Fig 3 shows methanol mole fraction in the reformers as a function of fuel feeding flow rate. Fuel flow rates of 0.1, 0.2 and 0.4 ml min^-1^ were simulated and compared. Accordingly, H_2_ flow rates for combustion are set as 60, 65, and 75 sccm. In Fig 3A, 3B and 3C, lowest methanol mole fraction at the outlet is found, which means the methanol is well consumed and a high methanol conversion shall be obtained. For each reactor, for example in Fig 3A, 3D and 3G, we can see that the methanol conversion gradually decreases as the fuel feeding rate rises. This can be explained that contact time for reactants and the catalyst bed is reduced when increasing the fuel flow speed. It is also observed that under the same condition of fuel feeding rate, the methanol conversion of tubular reactor is the lowest and cross-U type reactor is the highest. The average methanol mass fraction at the outlet of parallel-U type reactor increases from 0.2% to 9.5% and that of tubular reactor increases dramatically from 0.3% to 12.9%. In Fig 3C, 3F and 3I, methanol mass fraction at the outlet of cross-U type reactor increases from 0.2% to 8.7%. The smallest incensement indicates that cross-U type reactor has the best performance among the three reactors.

## Experimental

### Design, fabrication and measurement set-up

**Fig 4:**
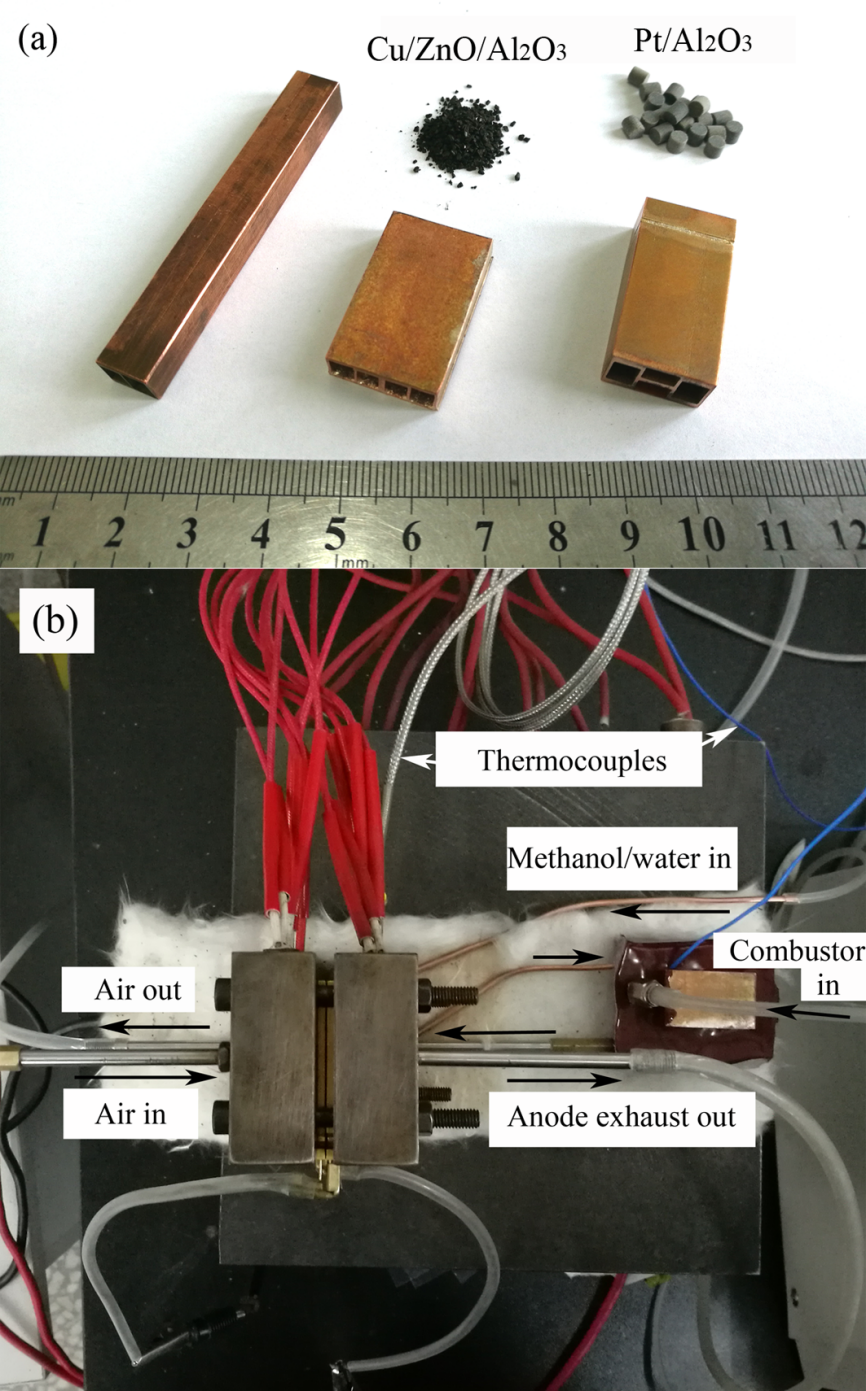
Photographs of (a) the reactors and catalysts used in the experiments, and (b) experimental set-up for stability test with HT-PEMFC.

As shown in Fig 4A, Cu-based micro reactors with the three structures were designed and fabricated. The length and channel size of the reactors were the same as the mathematical model. Precision machinery processing and vacuum brazing provided good welding and excellent air tightness for the reactors. Self-made Cu/ZnO/Al_2_O_3_ catalyst, which had a granular structure, was used in the reformer. Some of the main procedures were adopted from Jeong et al. [22].

The preparation of the reforming catalyst included 6 steps: (1) The metal nitrates, Cu(NO_3_)_2_, Zn(NO_3_)_2_, and Al(NO_3_)_3_ with a mole ratio of 2:1:1 were dissolved in de-ionized (DI) water with 1 M total metal concentration. (2) Aiming at pH = 8, Na_2_CO_3_ aqueous solution of 0.3 M was added with vigorous stirring and the protection of nitrogen at 60°C. (3) After 4 h of aging, the precipitates were filtrated and washed several times with DI water. (4) CuO/ZnO/Al_2_O_3_ catalyst was obtained after drying at 100°C for 12 h and calcined at 350°C for 4 h with a clean air stream. (5) The catalyst was reduced to Cu/ZnO/Al_2_O_3_ in a mixed stream of H_2_ (15%) and N_2_ at 350°C for 3 h. (6) Powdered catalyst was pressed to pellets, and then ball-milled to obtain the granular structure. Catalyst with the granular size amongst 0.7 and 1.7 mm was chosen with sieves. Commercially available Pt/Al_2_O_3_ pellets (Alfa Aesar, Product No. 89106) were used in the combustor for hydrogen and air combustion. Catalysts are also shown in Fig 4A. The temperature was sensed by a K-type thermocouple located on the outside wall of the reformer close to the inlet. Thermal insulator consisted of refractory fiber dope and acrylic latex coating was used in this study. The flow rates of hydrogen and air for combustion were controlled by gas flow controllers. Methanol solution was pre-heated and vaporized before feeding into reformer. Gas chromatograph was used to determine the mole fraction of H_2_, CO and CO_2_ in the reformed gas which was condensed and dried before measurements. In the stability test, the reformed gas was fed directly into a single cell HT-PEMFC. Fig 4B shows the experimental set-up for HT-PEMFC with the input of reformed gas. High temperature membrane electrolyte assembly (Advent TPS^®^) with an active area of 3×3 cm was used.

### Experimental results and discussion

**Fig 5:**
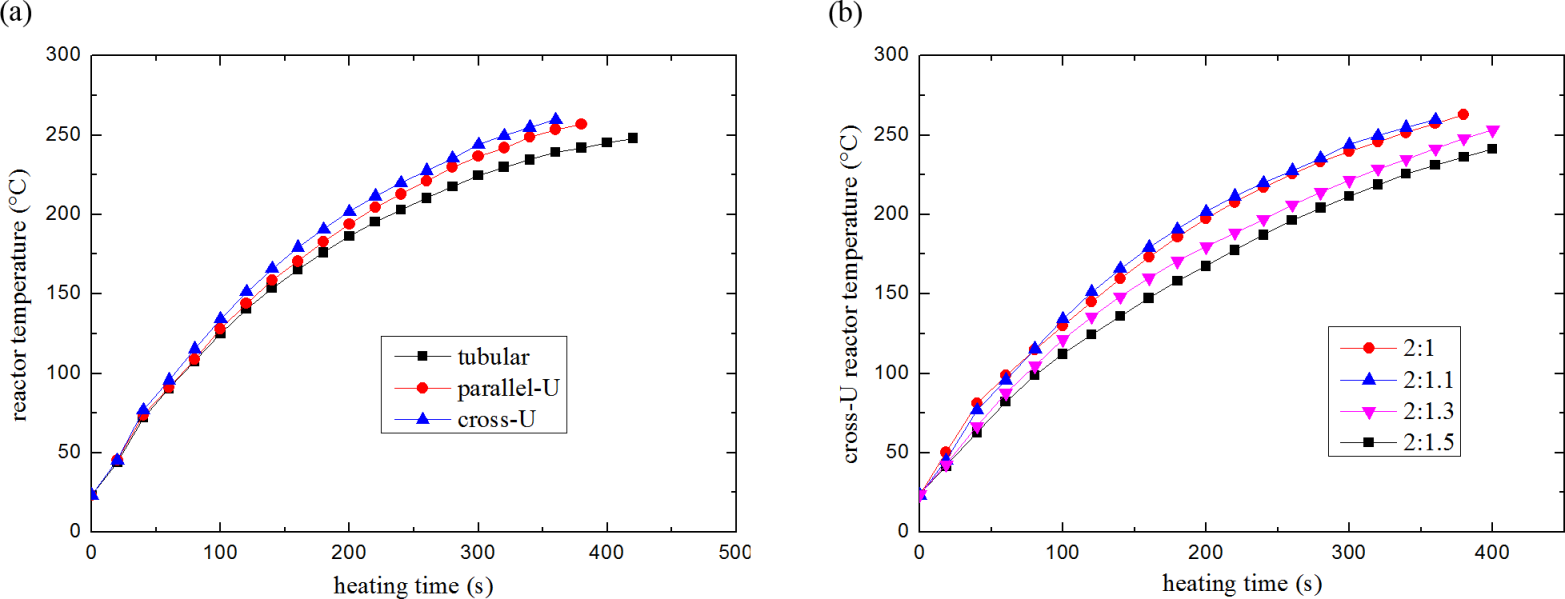
Combustor performance evaluation for each reactor: (a) reactor temperature as a function of heating time, and (b) effect of hydrogen to oxygen mole ratio.

Combustor performance was evaluated first. Fig 5A shows the temperature of the three reactors as a function of start-up time. For all the reactors, hydrogen flow rate was 80 sccm hydrogen and air flow rate was 300 sccm. Therefore, the hydrogen to oxygen mole ratio was about 5:3. It took less than 6 min for all the reactors to rise to 225°C. After testing several times, a slight advantage of cross-U type reactor was found. Compared to parallel-U type reactor, it took about 20 s less time for the cross-U type reactor to be thermally ready. The result was in good agreement with the simulation data shown in Fig 2 that cross-U type reactor has the best thermal performance. Fig 5B shows the effect of hydrogen to oxygen mole ratio (HOR) on the start-up time for cross-U type reactor. Various HORs of 2:1, 2:1.1, 2:1.3 and 2:1.5 were tested. The flow rates of H_2_ were kept at 80 sccm. The result indicates that HOR of 2:1.1 is the most appropriate for heating up the reactors. It can be explained by that at the same hydrogen supply rate, 10% excess air promoted the combustion reaction in the small reactor. Meanwhile, that was enough. More excess air just cooled down the reactor. Thus, HOR of 2:1.1 was regarded as the optimal ratio and was kept in the following experiments.

**Fig 6:**
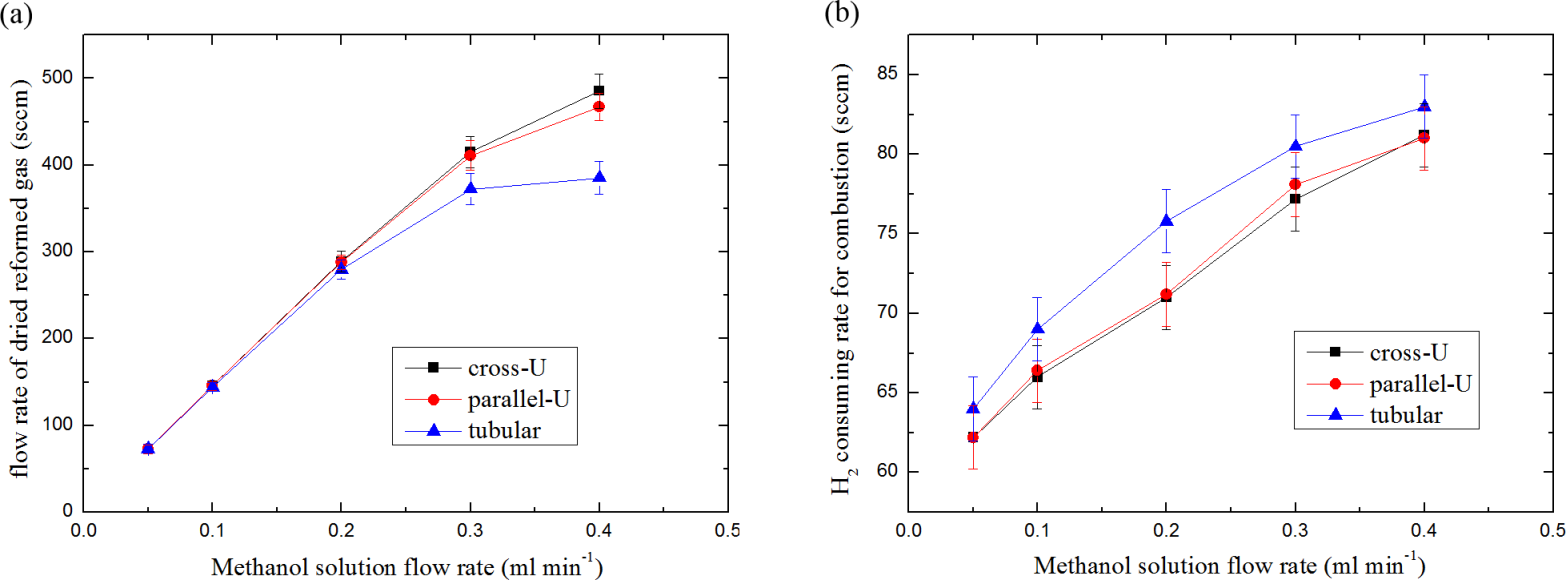
Reforming performance of the reactors at 240°C: (a) flow rate of dried reformed gas as a function of methanol solution feeding rate, and (b) the according H_2_ consuming rate for combustion.

After the optimal operating condition for combustor was determined, the reforming performance with various methanol solution feeding rates is summarized and shown in Fig 6. The H_2_ flow rates for combustion were dynamically controlled so that the temperature of reformers were kept at 240°C. Fig 6A shows the flow rates of reformed gas (dry base) with different methanol solution feeding rates. The flow rates of the reformed gas grow linearly with the fuel feeding rates in the relatively low range. The increasing tendency becomes smaller when the fuel feeding rate grows. It indicates the limits of the catalyst activity were reached. Obviously, cross-U type reactor shows the strongest ability of reforming under the temperature of 240°C. Fig 6B shows the according hydrogen consuming rate in the combustor. In attempting to maintain the reactor at 240°C, more hydrogen was consumed in tubular and parallel-U type reactors. Therefore, the energy efficiencies of the reactors are determined. With lowest H_2_ consuming rates in the combustor and highest reformed gas out of reformer, the cross-U type reactor clearly possesses the highest energy efficiency.

**Fig 7:**
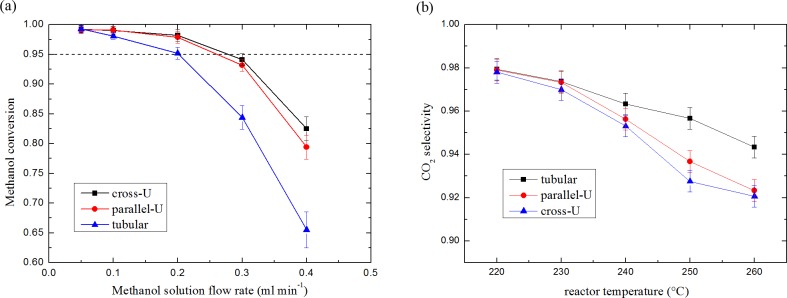
(a) Methanol conversion with different methanol solution feeding rate, and (b) CO_2_ selectivity under different temperatures for each reactor.

Fig 7A exhibits the methanol conversion of the reactors with different fuel flow rates at 240°C. Methanol conversion were calculated by:

$$\eta =\frac{1+\alpha }{3\alpha +2}\cdot \frac{f_{H_{2}}}{f_{\mathrm{MeOH}}}$$ (17)

where $f_{H_{2}}$ and *f*_MeOH_ represents the mole flow rates of reformed H_2_ and fed methanol, and *α* is the mole ratio of CO_2_ to CO, which was measured by gas chromatograph. The results reveal that the best performance was achieved by the cross-U type reactor at the flow rate of 0.1 ml min^-1^. On the contrary, the tubular exhibits the worst performance at the flow rate of 0.4 ml min^-1^. As a key factor affecting the performance of fuel cells based on reformed gas, methanol conversion over 95% is often required for reformers. Regarding to the threshold value, the cross-U type reactor produced the largest reformed gas (dry base) flow rate of 252.2 sccm, 23.5% higher than that of tubular reactor and 7.9% higher than parallel-U type reactor. CO_2_ selectivity of the reactors at different temperatures with the same fuel feeding rate of 0.2 ml min^-1^ are depicted in Fig 7B. The CO_2_ selectivity was calculated by:

$$s=\frac{\alpha }{1+\alpha }$$ (18)

The picture shows that cross-U type reactor had the lowest CO_2_ selectivity. It can be explained by the temperature distribution of the reactor. As mentioned above, the thermocouple was located on the outside wall of the reformer close to the inlet, which was demonstrated to be the hot spot for the reformer in the numerical simulation. Therefore, with the same temperature at the inlet and the most uniformly temperature distribution, cross-U type reactor had the highest overall temperature. Thus, more CO was generated in the cross-U type reactor than in the other two, especially tubular reactor.

**Fig 8:**
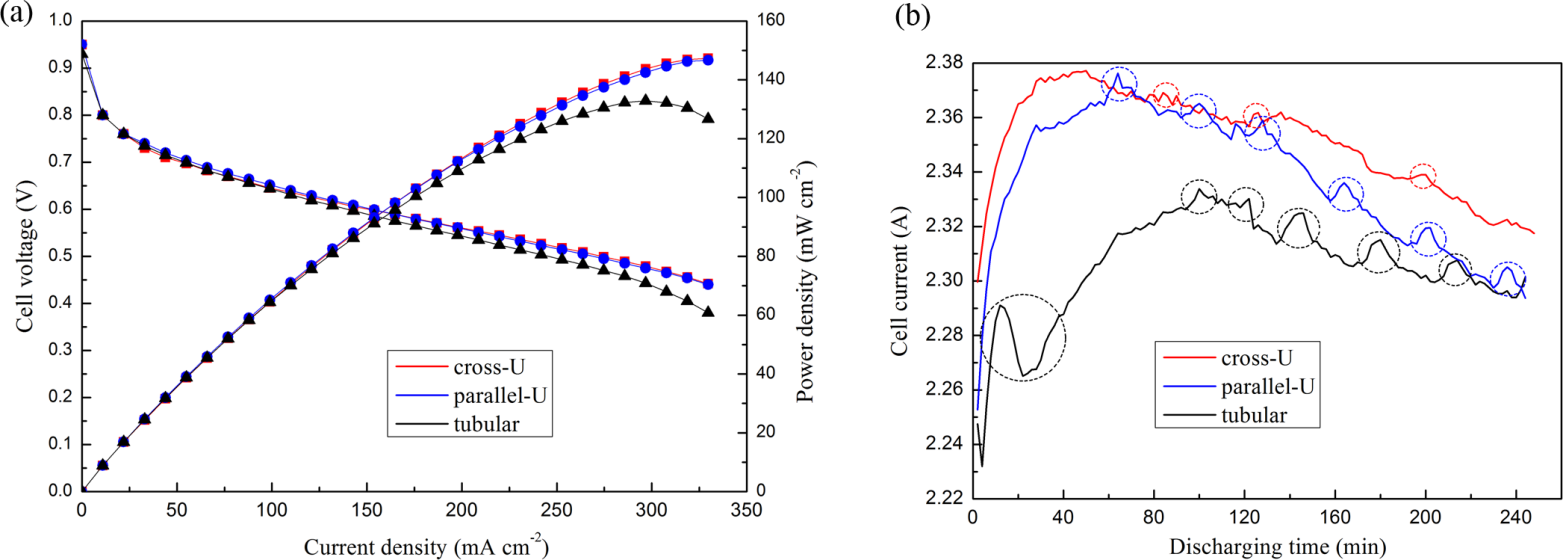
HT-PEMFC performance based on the reformed gas (a) polarization curve, and (b) discharging at constant 0.5 V for 4 h.

The performance of an HT-PEMFC fed with the reformed gas was evaluated as shown in Fig 8. The tests were conducted with the fuel cell under the temperature of 150°C. Flow rate of the methanol solution was kept at 0.1 ml min^-1^. It was seen from Fig 8A that the performance curves of the fuel cell for all reactors were very close when the discharging current was relatively low. However, when the current density was larger than 300 mA cm^-2^, the performance of the fuel cell fed by tubular reactor started to decrease. Fig 8B compares the stability of the discharging current of the fuel cell. The cell current was measured when the discharging voltage was constant at 0.5 V. Obviously, the performance for all the fuel cells fluctuated during the running for over 4 h. We consider this was mainly due to the following reasons. Firstly, the oscillations reflected the turbulences in the reformer, which could be caused by unstable reaction rate. Secondly, non-uniform distribution of temperature and peristaltic pump could intensify the effect. Thirdly, high concentration of unreacted methanol and carbon monoxide would poison the anode catalyst of the fuel cell. Nevertheless, as circled in the picture, the curve belonging to cross-U type reactor showed the slightest oscillations.

## Conclusions

In this study, a cross-U type micro reactor consisting of a reformer and a combustor for methanol steam reforming was presented and examined by numerical and experimental investigations. Firstly, a 3D CFD-based model coupled with mass transport, temperature distribution and chemical reactions was established. Secondly, Cu-based reactors were fabricated using precision machinery processing and vacuum brazing. Experiments were conducted on each reactor to test both combustion and reforming performance. Compared with conventional tubular and parallel-U type micro reactors, the new reactor structure exhibited higher thermal efficiency and methanol conversion. Simulation results were in good agreements with the experimental data. To guarantee a methanol conversion over 95% at 240°C, which is crucial for portable fuel cell application, cross-U type reactor was capable of yielding a hydrogen-enriched reformed gas of over 252.2 sccm. The flow rate was 8% and 23% higher than parallel-U type reactor and tubular reactor, respectively. Although higher temperature was beneficial for methanol steam reforming, the amount of CO in the reformed gas was also increased. In the measurements, CO fraction up to 2.4% was recorded in the dried reformed gas from the cross-U type reactor. It was then proved in this study that this amount of CO is acceptable for HT-PEMFCs. Furthermore, the fuel cell exhibited the most stable performance when fed with reformed gas from cross-U type reactor. Future work on highly integrated micro reformed methanol fuel cell system can be based on micro reformers from this study.
